# Ultrasonography Compared to Computed Tomography in Pediatric Complicated Pneumonia: Beyond Radiation

**DOI:** 10.7759/cureus.77158

**Published:** 2025-01-08

**Authors:** Purva Ajmire, Vidyanand Deshpande, Mohammad Haseeb, Madhurasree Nelanuthala, Simran Khanna

**Affiliations:** 1 Pediatrics, Mahatma Gandhi Mission (MGM) Medical College and Hospital, Aurangabad, IND; 2 Surgery, Mahatma Gandhi Mission (MGM) Medical College and Hospital, Aurangabad, IND; 3 Pediatrics and Neonatology, Mahatma Gandhi Mission (MGM) Medical College and Hospital, Aurangabad, IND

**Keywords:** hydropneumothorax, loculated pleural effusion, lung atelectasis, lung cavity, lung consolidation

## Abstract

Introduction

Some children with community-acquired pneumonia (CAP) experience progression to complicated community-acquired pneumonia (CCAP). It is characterized by local pulmonary or systemic complications such as para-pneumonic effusion, empyema, necrotizing pneumonia, and lung abscess. Imaging has an essential contribution to both the diagnosis and treatment of CCAP. While chest radiography and lung ultrasound (LUS) are commonly used for initial evaluation, chest computed tomography (CT), although valuable, raises concerns due to ionizing radiation exposure. Against the backdrop of increasing awareness regarding radiation risks in pediatric patients and the growing availability of bedside LUS, we conducted this study.

Objective

The aim of this study was to assess the agreement between LUS and contrast-enhanced chest CT in identifying specific findings in patients of complicated pneumonia, such as pleural effusion, pleural thickening, parenchymal consolidation, cavities, atelectasis, and hydropneumothorax.

Methods

We retrospectively compared CT and LUS images from 50 patients under 18 years of age admitted with clinical and radiological diagnosis of complicated pneumonia between January 2022 and June 2023, who underwent both imaging modalities within a seven-day interval. Images were assessed for pleural effusion, septations, pleural thickening, parenchymal consolidation, cavities, hydropneumothorax, and atelectasis.

Results

Pleural effusion without septations was found in 25 (50%) patients on chest CT, whereas it was noted in 20 (40%) patients on LUS. Pleural effusion with septations was found in 21 (42%) patients on chest CT, whereas it was noted in 27 (54%) patients on LUS. Pleural thickening was found in 19 (38%) patients on chest CT, whereas it was noted in 9 (18%) patients on LUS. Lung parenchymal consolidation was found in 30 (60%) patients on chest CT, whereas it was noted in 16 (32%) patients on LUS. Atelectasis was found in 25 (50%) patients on chest CT, whereas it was noted in 26 (52%) patients on LUS. Parenchymal cavities were found in six (12%) patients on chest CT, whereas it was noted in two (4%) patients on LUS. Hydropneumothorax was found in six (12%) patients on chest CT, whereas it was noted in one (2%) patients on LUS.

Conclusions

LUS can be used as an initial investigation in complicated pneumonia, while chest CT is reserved for cases with suspicion of specific complications of pneumonia.

## Introduction

Community-acquired pneumonia (CAP) in children is a significant cause of morbidity and mortality worldwide, accounting for a substantial burden on healthcare systems. Despite advances in preventive and therapeutic strategies, CAP remains a leading infectious cause of death in children under five years of age in many regions, particularly in low- and middle-income countries [1, 2]. Among these cases, complicated pneumonia represents a severe subset, characterized by para-pneumonic effusion, parenchymal consolidation, cavities, or lung atelectasis. The rising prevalence of complicated pneumonia in pediatric populations highlights the need for timely and accurate diagnostic tools to optimize patient outcomes [3,4]. Radiographic imaging has long been the cornerstone of diagnosing and managing complicated CAP. Chest computed tomography (CT) is widely recognized as the gold standard for identifying advanced parenchymal disease and associated complications. However, the reliance on CT raises several challenges. CT imaging exposes children to ionizing radiation, which has potential long-term health implications, especially in younger patients. Moreover, logistical issues, such as the need to transport critically ill children to radiology centers, further complicate its routine use in pediatric care [5-9]. These concerns have prompted the exploration of alternative, safer imaging modalities.

Lung ultrasound (LUS) has emerged as a promising diagnostic tool for evaluating complicated pneumonia in children. Its advantages include the absence of ionizing radiation, bedside applicability, and cost-effectiveness, making it particularly useful in resource-limited settings. Despite these benefits, there remains limited consensus on whether LUS can replace chest CT in the diagnostic algorithm for complicated pneumonia [5]. The lack of standardized guidelines and variable operator dependency have hindered its widespread adoption. With this perspective, the present retrospective study was conducted to assess the agreement between LUS and contrast-enhanced CT in identifying specific findings of complicated pneumonia, such as pleural effusion, pleural thickening, parenchymal consolidation, cavities, atelectasis, and hydropneumothorax.

## Materials and methods

The present retrospective study was conducted at the Department of Pediatrics, MGM Medical College and Hospital, Aurangabad, Maharashtra, India. The institutional Ethics Committee approved the study protocol prior to commencement of this study. Informed consent was obtained from the parents or legal guardian of the study participants. The study compared chest CT and LUS reports from 50 patients under 18 years of age, who were admitted with clinical and radiological diagnoses of complicated pneumonia between January 2023 and February 2024 and underwent both imaging modalities within a seven-day interval.

An experienced radiologist performed LUS using GE Voluson S10 (GE Healthcare, Milwaukee, WI, USA) and GE Voluson E8 (GE Healthcare) and chest CT using Fujifilm Supria 32 slice (Fujifilm Corporation, Tokyo, Japan). The LUS and CT images were examined for the presence of pleural effusion, septations, pleural thickening, parenchymal consolidation, cavities, atelectasis, and hydropneumothorax.

Inclusion criteria

The study included patients under 18 years of age with a clinical and radiological diagnosis of complicated pneumonia, patients who underwent both chest CT and LUS within a seven-day interval during their hospitalization, patients with complete medical records including detailed imaging reports for both modalities, and patients with consent from parent or guardian for participation in the study.

Exclusion criteria

The study excluded patients with pre-existing chronic pulmonary diseases or structural lung abnormalities unrelated to pneumonia and patients who underwent surgical intervention for pneumonia-related complications before imaging.

The findings on chest CT and lung USG with respect to medical diagnosis in cases of pleural effusion, septations, pleural thickening, parenchymal consolidation, cavities, atelectasis, and hydropneumothorax were described. The number and percentage of cases that were consistent with the final diagnosis on LUS or chest CT as pleural effusion, septations, pleural thickening, parenchymal consolidation, cavities, atelectasis, and hydropneumothorax were described.

## Results

A total of 50 patients were enrolled for the study, of which 33 were males and 17 were females. The mean age of the study participants was 7 ± 5 years. LUS was performed first, and the mean time between LUS and CT was 3.7 days (range, 0-7 days). Table 1 describes the age distribution of study participants.

**Table 1:** Age distribution of study subjects

Age	Number of patients	Percentage
0-1 year	5	10%
>1 year to 5 years	18	36%
>5 years to 18 years	27	54%
Total	50	100%

LUS demonstrated superior detection of septations within pleural effusions. Table 2 describes the findings consistent with the final diagnosis on chest CT compared with LUS in septate pleural effusion.

**Table 2:** Findings consistent with the final diagnosis on chest CT compared with LUS in septate pleural effusion CT, computed tomography; LUS, lung ultrasound

Findings	Chest CT	Lung Ultrasound
Septate pleural effusion	21 (42%)	27 (54%)

Chest CT was better in detection of pleural thickening, lung consolidation, pulmonary cavities, and hydropneumothorax. Table 3 describes the findings consistent with final diagnosis on chest CT compared with LUS in pleural thickening, lung consolidation, pulmonary cavities, and hydropneumothorax.

**Table 3:** Findings consistent with final diagnosis on chest CT compared with LUS in pleural thickening, lung consolidation, pulmonary cavities, and hydropneumothorax CT, computed tomography; LUS, lung ultrasound

Findings	Chest CT	Lung Ultrasound
Pleural thickening	19 (38%)	9 (18%)
Lung consolidation	30 (60%)	16 (32%)
Pulmonary cavities	6 (12%)	2 (4%)
Hydropneumothorax	6 (12%)	1 (2%)

Chest CT and LUS were similar in performance in detecting non-septate pleural effusion and atelectasis. Table 4 describes the findings consistent with final diagnosis on chest CT compared with LUS in non-septate pleural effusion and atelectasis.

**Table 4:** Findings on chest CT compared with LUS in non-septate pleural effusion and atelectasis CT, computed tomography; LUS, lung ultrasound

Findings	Chest CT	Lung Ultrasound
Non-septate pleural effusion	25 (50%)	20 (40%)
Atelectasis	25 (50%)	26 (52%)

**Figure 1:**
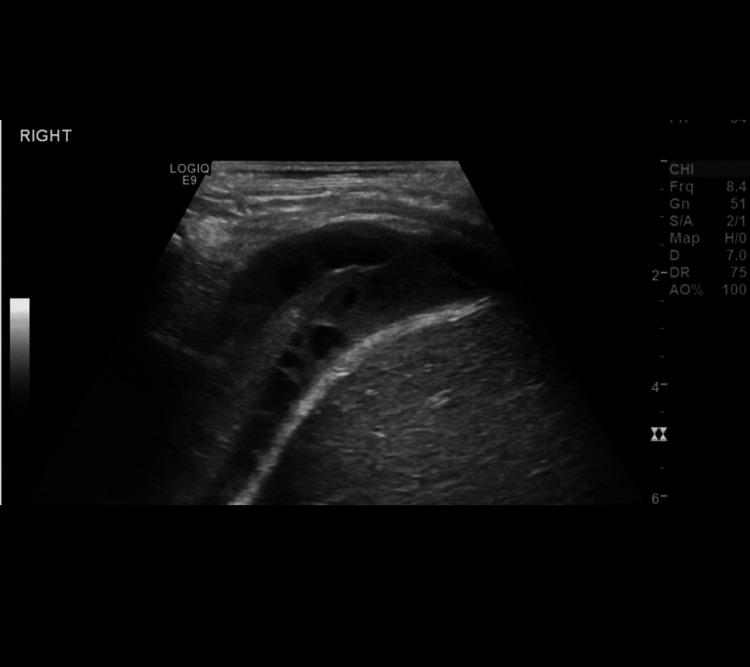
Ultrasound examination in pleural effusion with septations

Figure 1 shows the image of targeted ultrasound examination of the right hemithorax, demonstrating a complex collection in the pleural space. The collection has heterogenous internal echoes and septations, without vascularity, consistent with empyema. The adjacent right basal lung is consolidated/collapsed.

**Figure 2:**
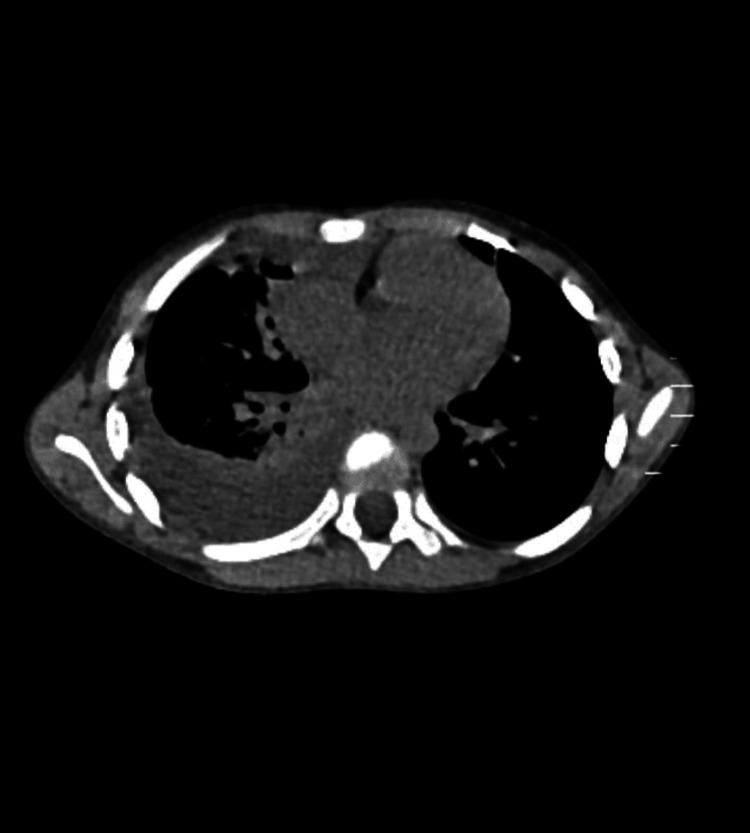
CT image of the loculated collection in the left pleural cavity

Figure 2 shows the CT image of the loculated collection in the left pleural cavity.

## Discussion

Chest CT was better in detection of pleural thickening, lung consolidation, pulmonary cavities, and hydropneumothorax. LUS performance was comparable to the chest CT in detecting pleural effusion without septations and atelectasis. LUS performed better in detection of septate pleural effusion. The better performance of chest CT in lung consolidation and pulmonary cavities has clinical significance as it impacts management decisions [10].

The British Thoracic Society recommended LUS to assess pleural effusion; however, chest CT is better for the evaluation of lung parenchymal disease [7]. Kurian et al. reported that LUS was similarly effective in the diagnosis of effusion, lung consolidation, and other findings in complicated pneumonia. They found no significant additional information provided by chest CT beyond the clinically relevant insights obtained by LUS for the evaluation of complicated pneumonia [5]. The utility of LUS in the detection of septate pleural effusion has been supported by earlier studies [9,11,12].

Further studies reinforce the value of LUS in clinical practice. For example, the similar performance of LUS in detecting pleural effusion, combined with its advantages such as lower cost, lack of ionizing radiation, and bedside availability, has been emphasized by various researchers [13-18]. Sharma and Farahbakhsh reported the discriminating ability of LUS in atelectasis and pneumothorax, which further highlights its utility in acute settings [19]. Additionally, Carrard et al. observed that LUS was effective in diagnosing necrotizing pneumonia [20].

Our findings align with those of Amatya et al., who reported that LUS remains a preferred tool for bedside evaluations in pediatric pneumonia due to its non-invasive nature and real-time diagnostic capability [21]. Moreover, the role of LUS in guiding thoracentesis and other interventional procedures in pediatric patients has been increasingly acknowledged [22,23].

Despite these advantages, it is essential to recognize the limitations of LUS, including operator dependency and limited field of view in certain clinical scenarios. The complementary roles of LUS and chest CT in specific cases, such as in the evaluation of complex or atypical presentations, underscore the importance of a tailored approach to imaging in pediatric respiratory diseases. Future research could focus on refining LUS protocols and training to optimize its utility in diverse healthcare settings.

Limitations

The study has a small sample size from a single center and a retrospective study design. The variability of findings by radiologists has not been taken into consideration.

## Conclusions

LUS offers a valuable alternative to chest CT in evaluating complicated pneumonia in children. It is a bedside, radiation-free, rapid, non-invasive, cost-effective, and easily repeatable tool. These features make it particularly advantageous for pediatric care, especially in resource-limited settings.

However, chest CT remains essential when ultrasound findings are inconsistent with clinical presentations or when specific complications, such as abscesses or cavities, are suspected. Integrating LUS into routine practice can complement chest CT, enhancing the diagnostic approach to managing complicated pneumonia.

## References

[1] GBD 2016 Lower Respiratory Infections Collaborators (2018). Estimates of the global, regional, and national morbidity, mortality, and aetiologies of lower respiratory infections in 195 countries, 1990-2016: a systematic analysis for the Global Burden of Disease Study 2016. Lancet Infect Dis.

[2] McIntosh K (2002). Community-acquired pneumonia in children. N Engl J Med.

[3] de Benedictis FM, Kerem E, Chang AB, Colin AA, Zar HJ, Bush A (2020). Complicated pneumonia in children. Lancet.

[4] Tan TQ, Mason EO Jr, Wald ER (2002). Clinical characteristics of children with complicated pneumonia caused by Streptococcus pneumoniae. Pediatrics.

[5] Kurian J, Levin TL, Han BK, Taragin BH, Weinstein S (2009). Comparison of ultrasound and CT in the evaluation of pneumonia complicated by parapneumonic effusion in children. AJR Am J Roentgenol.

[6] Buonsenso D, Brancato F, Valentini P, Curatola A, Supino M, Musolino AM (2020). The use of lung ultrasound to monitor the antibiotic response of community-acquired pneumonia in children: a preliminary hypothesis. J Ultrasound Med.

[7] Balfour-Lynn IM, Abrahamson E, Cohen G (2005). BTS guidelines for the management of pleural infection in children. Thorax.

[8] Brenner D, Elliston C, Hall E, Berdon W (2001). Estimated risks of radiation-induced fatal cancer from pediatric CT. AJR Am J Roentgenol.

[9] Yang L, Wang K, Li W, Liu D (2024). Chest ultrasound is better than CT in identifying septated effusion of patients with pleural disease. Sci Rep.

[10] Calder A, Owens CM (2009). Imaging of parapneumonic pleural effusions and empyema in children. Pediatr Radiol.

[11] Kim OH, Kim WS, Kim MJ, Jung JY, Suh JH (2000). US in the diagnosis of pediatric chest diseases. Radiographics.

[12] Chiu CY, Wong KS, Huang YC, Lai SH, Lin TY (2006). Echo-guided management of complicated parapneumonic effusion in children. Pediatr Pulmonol.

[13] Soni NJ, Franco R, Velez MI, Schnobrich D, Dancel R, Restrepo MI, Mayo PH (2015). Ultrasound in the diagnosis and management of pleural effusions. J Hosp Med.

[14] El-Sayed Ibrahim El-Sayed I, Farouk Awad N, Shawky Basiony F, Salah El-Feshawy M (2022). Chest ultrasound versus chest computed tomography for assessment of undiagnosed pleural effusion before medical thoracoscopy. Al-Azhar Med J.

[15] Iovine E, Nenna R, Bloise S (2021). Lung ultrasound: its findings and new applications in neonatology and pediatric diseases. Diagnostics (Basel).

[16] Reissig A, Copetti R, Mathis G (2012). Lung ultrasound in the diagnosis and follow-up of community-acquired pneumonia: a prospective, multicenter, diagnostic accuracy study. Chest.

[17] Claes AS, Clapuyt P, Menten R, Michoux N, Dumitriu D (2017). Performance of chest ultrasound in pediatric pneumonia. Eur J Radiol.

[18] Konietzke P, Mueller J, Wuennemann F (2020). The value of chest magnetic resonance imaging compared to chest radiographs with and without additional lung ultrasound in children with complicated pneumonia. PLoS One.

[19] Sharma D, Farahbakhsh N (2019). Role of chest ultrasound in neonatal lung disease: a review of current evidences. J Matern Fetal Neonatal Med.

[20] Carrard J, Bacher S, Rochat-Guignard I (2023). Erratum: Necrotizing pneumonia in children: chest computed tomography vs. lung ultrasound. Front Pediatr.

[21] Amatya Y, Russell FM, Rijal S, Adhikari S, Nti B, House DR (2023). Bedside lung ultrasound for the diagnosis of pneumonia in children presenting to an emergency department in a resource-limited setting. Int J Emerg Med.

[22] Singh Y, Tissot C, Fraga MV (2020). International evidence-based guidelines on Point of Care Ultrasound (POCUS) for critically ill neonates and children issued by the POCUS Working Group of the European Society of Paediatric and Neonatal Intensive Care (ESPNIC). Crit Care.

[23] Ammirabile A, Buonsenso D, Di Mauro A (2021). Lung ultrasound in pediatrics and neonatology: an update. Healthcare (Basel).

